# Major Histocompatibility Complex (MHC) Genes and Disease Resistance in Fish

**DOI:** 10.3390/cells8040378

**Published:** 2019-04-25

**Authors:** Takuya Yamaguchi, Johannes M. Dijkstra

**Affiliations:** 1 Laboratory of Fish Immunology, Friedrich-Loeffler-Institute, Boddenblick 5A, 17498 Insel Riems, Germany; Takuya.Yamaguchi@fli.de; 2 Institute for Comprehensive Medical Science, Fujita Health University, Toyoake, Aichi 470-1192, Japan

**Keywords:** fish, MHC, polymorphism, disease resistance, quantitative trait loci (QTL) studies, evolution

## Abstract

Fascinating about classical major histocompatibility complex (MHC) molecules is their polymorphism. The present study is a review and discussion of the fish MHC situation. The basic pattern of MHC variation in fish is similar to mammals, with MHC class I versus class II, and polymorphic classical versus nonpolymorphic nonclassical. However, in many or all teleost fishes, important differences with mammalian or human MHC were observed: (1) The allelic/haplotype diversification levels of classical MHC class I tend to be much higher than in mammals and involve structural positions within but also outside the peptide binding groove; (2) Teleost fish classical MHC class I and class II loci are not linked. The present article summarizes previous studies that performed quantitative trait loci (QTL) analysis for mapping differences in teleost fish disease resistance, and discusses them from MHC point of view. Overall, those QTL studies suggest the possible importance of genomic regions including classical MHC class II and nonclassical MHC class I genes, whereas similar observations were not made for the genomic regions with the highly diversified classical MHC class I alleles. It must be concluded that despite decades of knowing MHC polymorphism in jawed vertebrate species including fish, firm conclusions (as opposed to appealing hypotheses) on the reasons for MHC polymorphism cannot be made, and that the types of polymorphism observed in fish may not be explained by disease-resistance models alone.

## 1. Introduction

### 1.1. The Polymorphism of MHC Genes

Extensive gene polymorphism (variation between alleles) is unusual because in situations in which an allele is superior to all others or if alleles are neutral, selection or chance occurrence induces allele fixation throughout the population [1,2]. To maintain polymorphism as found for classical MHC, a process called “balancing selection” is necessary, which means there is evolutionary pressure favoring allelic variation within a species [3,4]. Classical MHC genes were reported to be the most polymorphic genes in the human genome [5,6], and extensive classical MHC polymorphism is a common feature among jawed vertebrate species [7]. Long before the responsible genes were identified, phenotypic effects of genetic MHC variation were already known because of rejection of MHC-mismatched (“HLA”-mismatched in human; “H2”-mismatched in mice) allogeneic tissue and cell grafts in humans and mice (reviewed by [8]). Because several of the responsible genes are linked together in a gene-“complex” region, which shows similarity between humans and mice, the genomic region was called the Major Histocompatibility Complex (in this article *Mhc* is used when referring to the region), and the family of genes encoding the targets for allogeneic rejection were called (classical) MHC genes. The later elucidation of the function of MHC molecules in peptide antigen presentation to T cells [9], and the effects of MHC polymorphism on that peptide presentation [10], led many researchers to believe that MHC-mediated allograft rejection was only an artifact, as stated by Michalová et al. in 2000 ([11]; the Jan Klein group): “The allograft reaction is an artifactual manifestation of the true function of the class I and class II loci, which is the presentation of antigenic peptides for recognition by T lymphocytes and thus the initiation of the adaptive immune response.” However, although the models explaining MHC polymorphism based on disease resistance variation are intellectually appealing (see below), there has been little experimental support. In 1994, Satta et al. ([12]; the Jan Klein group) stated: “…it has proved difficult to demonstrate the presence of balancing selection at MHC loci experimentally. Only a few cases of associations between specific MHC alleles and resistance to parasites in natural populations have been reported, and even these are not entirely convincing.” Not much has changed since then, despite more than 20 years of research including many more species, with Kelly and Trowsdale stating in 2017 [13]: “It is widely assumed that resistance to infection is driving the extreme MHC variation, although direct evidence for this is limited.” The word limited in this citation refers to the fact that only very few cases have been reported that rather convincingly show correlations between MHC sequence polymorphism and pathogen resistance (see below), and that even if those few correlations are true they may be considered “anecdotal evidence” instead of final proof for the “pathogen resistance model”. This is not to say that the model is not true, as it has been pointed out that even small advantages that are very hard to capture experimentally can lead to evolutionary selection [12]. Nevertheless, researchers should probably keep an open mind for the possibility of other or additional explanations for MHC polymorphism. An important part of this review considers the fact that in many fish species the observed polymorphism in classical MHC class I shows features which cannot easily be explained by the pathogen resistance model, and that other evolutionary pressures may (additionally) shape fish MHC polymorphism. The present review is a summary of what is currently known about fish MHC, with a focus on polymorphism and disease resistance.

### 1.2. MHC Variation and Resistance to Infectious Diseases in Tetrapod Species

Classical MHC class I and II molecules present peptide antigens for recognition by T cell receptors (TCR) on CD8^+^ cytotoxic and CD4^+^ helper/regulatory T cells, respectively [9,14]. The classical MHC class I and II molecules show extensive allelic polymorphism in residues lining the peptide binding groove, which leads to a presentation of different sets of antigen peptides by different individuals of the same species [10,15]. By comparing synonymous and nonsynonymous nucleotide exchange rates, many of the MHC residues lining the peptide binding groove have been shown to be under evolutionary selection towards sequence variation [16,17]. Within species allelic MHC variation tends to partially predate the most recent speciation events (is inherited from ancestral species), reflected in trans-species allelic sequence lineages, while also new allelic variation keeps being generated by point mutation and/or recombination events supposedly driven by a race of arms with pathogens [18,19]. The allelic MHC variation is commonly believed to increase the protection of a population of species against pathogens that otherwise might more easily evade effective MHC presentation by all individuals in a population (reviewed in [20]). However, differences in resistance to infectious diseases have rarely been strongly linked to *Mhc* haplotypes (see above [12,13,20,21]). Genotype–phenotype linkage studies investigating potential linkage of MHC genotypes with differences in pathogen resistance cannot easily differentiate between effects caused by differences in the MHC peptide binding groove, by other variable features of the MHC molecules (e.g., expression levels), or by variation in linked genes. The most compelling case for explaining differences in resistance to an infectious disease by different MHC alleles presenting different sets of peptides probably has been made for resistance and susceptibility to HIV conferred by certain HLA-B molecules (reviewed in [22]).

### 1.3. MHC in Fish

Although in teleost fishes (modern bony fishes) allograft rejection and thymus-dependent antibody responses had been known for quite a while (reviewed in [23]), it was only in 1990 that the first fish MHC class I and II genes were found in common carp [24]. Later, MHC class I [25] and class II [26] were found in cartilaginous fish, followed by the detection of MHC class I and II in lobe-finned fishes [27,28,29]. Jawless fish and invertebrates do not have MHC genes [30]. There now have been a number of reports on genomic organizations of fish MHC genes, on their polymorphism and their expression, and there have also been some reports on teleost MHC at the protein and functional level. Those studies are summarized in the next paragraphs. In short, although the evidence is fragmentary, the functions of classical fish MHC class I and II molecules in fish appear to be similar to those in mammals. Table 1 provides an overview comparing MHC (system) traits in teleost fish and mammals.

### 1.4. Associations between MHC Variation and Differences in Disease Resistance in Fish

Some fish species, for example Salmoniformes, can have large numbers of offspring in single broods (sometimes >1000), which is helpful for genotype–phenotype linkage association studies. In regard to linkage analysis of teleost fish genotypes with disease resistance there have been studies specifically dedicated to MHC genes, as well as genome-wide quantitative trait loci (QTL) studies that allow investigation of the impact of linkage groups harboring MHC genes. The present article focuses on the summary of those QTL reports, while largely neglecting the genotype–phenotype studies specifically dedicated to MHC genes. The reason for this is that, generally, we only deem the QTL analyses as sufficiently objective and trustworthy from human behavior and statistical points of view; most genotype–phenotype studies dedicated to fish MHC genes suffer from the statistical modeling weakness that no predictions were made, and we deem it psychologically risky to give (mostly young) researchers tasks that only result in publications if they do find some interesting associations. At a note, in the past the last author (J.M.D.) has been involved in a project partially funded to find differences in anti-virus disease resistance associated with the highly polymorphic classical MHC class I locus in rainbow trout, but could not find such differences (unpublished data). From that project, however, he learned that such negative data tend not to make it into publications (worsening the statistical integrity of the body of published articles) and that there is an enormous career-pressure to present (part of) the data from an (artificial, outcome-selected) angle that would suggest statistical relevance. Luckily, nowadays there have been a number of QTL studies in fish providing the luxury of being able to rely only on those studies for the main conclusions on genotype–phenotype associations. The body of published QTL studies provides no indications that the enormous allelic variation in teleost fish classical MHC class I variation causes differences in disease resistance, but might hint at possible influences of MHC class II allelic variation and of nonclassical MHC class I intact allele versus null-allele variation.

## 2. Fish Phylogeny

**Figure 1:**
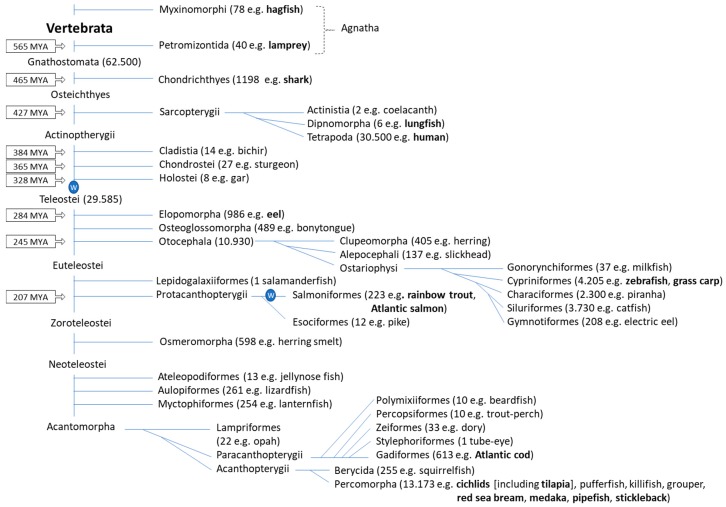
Schematic overview of fish classification. The organization of fish clades, and the estimated number of species which is indicated between brackets for some of them, are based on Nelson et al., 2016 [96]. Also between brackets, simplified popular English names of fish species representative for the clade are given; fish species that are important in the main text are highlighted in bold. For some nodes the time of separation in million years ago (MYA) is given as calculated in [98]. Circles with the letter W refer to whole genome duplication events early in the lineages Teleostei and Salmoniformes. Fish phylogeny and species numbers are under continued discussion, and the figure should be understood as an approximation.

When discussing fish MHC, it is important to realize that this is a phylogenetically hugely diversified group. Fishes constitute approximately half of all vertebrate species [96], and can be divided into multiple clades (Figure 1). The most primitive extant fish are the Agnatha (“no jaws”), which include the extant lamprey and hagfish, and which have an immune system without MHC and TCR [30]. Although the precise timings are a matter of debate, the lineage separation of the Agnatha and the Gnathostomata (“jawed mouth”) probably occurred around 565 million years ago (MYA), after which the Chondrichthyes (cartilaginous fish like shark and ray) separated from the Osteichthyes (bony fish) around 465 MYA, followed by the separation between Sarcopterygii (lobe-finned fish and tetrapods) and Actinopterygii (ray-finned fish) around 427 MYA [97,98]. The immune system throughout Gnathostomata appears to be basically similar, with an important role for MHC molecules [30]. The Teleostei (teleosts) constitute the vast majority of the extant Actinopterygii (Figure 1; [96]), and their genomes are characterized by remnants of a whole genome duplication (WGD) event that happened early in the teleost lineage [99]. Because of their abundance and economical importance, the teleosts are the most intensively investigated fish group. Three important teleost clades are the Otocephala (with e.g., herring, catfish, and Cypriniformes like grass carp and zebrafish), the Protacanthopterygii (with e.g., Salmoniformes like rainbow trout and Atlantic salmon) and the Neoteleostei (neoteleosts, e.g., cichlids, pufferfish, medaka, stickleback, cod). In teleost fish, the most comprehensive MHC work has probably been done on Salmoniformes (e.g., rainbow trout and Atlantic salmon) and Cypriniformes (e.g., zebrafish and grass carp), whereas for Neoteleostei, which comprise the biggest group of teleost fish (Figure 1), high quality MHC genetic analyses were performed for medaka.

## 3. Classical and Nonclassical MHC Genes

### 3.1. Classical and Nonclassical MHC Class II

In mammals, classical MHC class II molecules show extensive allelic polymorphism and are expressed at the surface of professional antigen presenting cells (APCs; for example, B-cells and macrophages) where they have an important immune function in presenting peptides from endocytosed/phagocytosed antigens to TCRαβ^+^CD4^+^ T lymphocytes [9,14]. In humans, the classical MHC class II molecules are HLA-DP, -DQ, and -DR, while the non-classical MHC class II molecules are HLA-DM and -DO. The HLA-DM and HLA-DO molecules do not present peptides but have a “peptide-editing” (helping to select for high affinity peptides) function in the peptide loading system of classical MHC class II [100]. Whereas HLA-DO appears to be an evolutionary relatively young diversification from the classical MHC class II lineage, DM lineage genes can already be found in lungfish and DM lineage is stably inherited throughout tetrapod species [27,67]. Despite absence of DM, teleost MHC class II molecules can be distinguished into classical versus nonclassical based on polymorphism, expression patterns, and presence/absence of residues important for peptide ligand binding (e.g., [27,35,36,37,38]). The first convincing descriptions of classical MHC class II polymorphism in fish probably were for zebrafish by Ono et al., 1992 [39], and nurse shark by Kasahara et al., 1993 [101]. The old nonclassical MHC class II lineages found in teleost fish were named “DB” and “DE”, but unlike the DM lineage in tetrapods these lineages are not stably inherited throughout most teleosts [27]. Whereas indirect evidence indicates the presence of classical MHC class II functions in fish, there are no good clues for allowing speculation on the functions of nonclassical teleost MHC class II. In teleost fish, in many cases, the nonclassical and classical MHC class II genes are not linked with each other [27].

### 3.2. Classical and Nonclassical MHC Class I

In mammals, classical MHC class I molecules show extensive allelic polymorphism and are expressed at the surface of most nucleated cells where they have an important immune function in presenting peptides from intracellular antigens to TCRαβ^+^CD8^+^ T lymphocytes [9,14]. In addition, tetrapod species have a wide variety of nonclassical MHC class I molecules, which are not stably inherited among species clades [40,102], do not show the polymorphism of the classical molecules, and have a wide variety of functions within and outside the immune system [103].

The first description of a fish gene encoding a classical MHC class I molecule, identified by the peptide termini binding residues (reviewed in [41]) and in later studies by extensive allelic polymorphism, was by Grimholt et al., 1993 [42], for Atlantic salmon. Like in tetrapod species, most of the well-investigated teleost species have one, two or three polymorphic classical MHC class I genes per haploid genome that encode proteins with a (predicted) conserved ability to bind peptides, and a variable number of nonclassical genes that are closely related to these classical genes (e.g., [49,50,104,105]); together these teleost molecules have been assigned as the “U lineage” [43]. Besides the U lineage genes, in teleost fish also genes of diverged nonclassical MHC class I lineages now named Z [24,44], S [106], L [45], and P [43] can be found. The S, L and P lineages are not stably inherited throughout teleosts, and their function is unknown [43]. The Z lineage is divided into “typical” and “atypical” molecules, with the typical Z molecules probably representing the more original form which is found in all investigated teleosts but also in spotted gar, bichir and lungfish [29,40,43,44,107]. The “atypical” Z molecules constitute highly differentiated Z forms and are only found in some teleost species [24,43]. The function of typical Z molecules is not known, but comparison with sequence motifs of classical MHC class I suggests that they bind conserved peptides of approximately 8-9 amino acids with a modification of the N-terminus. The conservation among the residues estimated to line the Z binding groove is near-absolute between species as widely divergent as lungfish, gar, bichir and teleosts [40,43], which is unprecedented among MHC molecules. The number of genes for typical Z molecules in different fish species can differ between 1 and ~10 [43,107], and within species considerable differences in their expression patterns and encoded features like cytoplasmic tails are observed [43,44,107]. Like in mammalian genomes, in teleost fish genomes the classical MHC class I genes tend to be linked with each other and in many cases they are also linked with some nonclassical MHC class I genes, whereas other nonclassical MHC class I genes are dispersed over the genome [43,105,108].

## 4. Allelic Variation in Classical MHC Molecules in Fish

### 4.1. Allelic Variation in Fish Classical MHC Class II

The important study by Shum et al., 2001 [46], concluded that in investigated fish species, similar as in tetrapods, there has been an evolutionary selection towards within species variation (balancing selection) in those residues of classical MHC class I and class II molecules that line the peptide binding groove and (probably) influence peptide preferences. The types of allelic variation in classical MHC class I and II in mammals and sharks, and classical MHC class II in teleost fish, seem to be relatively similar, and appear to be mostly dedicated to creating variations in the peptide binding groove that (are predicted to) affect the selection of bound peptides (e.g., [46,109,110]). There now have been quite a number of reports on classical MHC class II variation within teleost fish species, but it is not always clear from which gene locus (or how many loci) the variable gene sequences are derived (e.g., see Text S3 in [27]). Convenient for interpretations of classical MHC class II sequence variation is the situation in species like Atlantic salmon and rainbow trout, because these salmonid species have only one classical MHC class II locus with one gene for an alpha chain and one gene for a beta chain [46,109,111]. Interestingly, whereas in tetrapods the classical MHC class II alpha chains tend to show little allelic variation, in Salmoniformes the alpha and beta chain allelic variations appear to be similarly extensive [109].

### 4.2. Allelic Variation in Fish Classical MHC Class I

**Figure 2:**
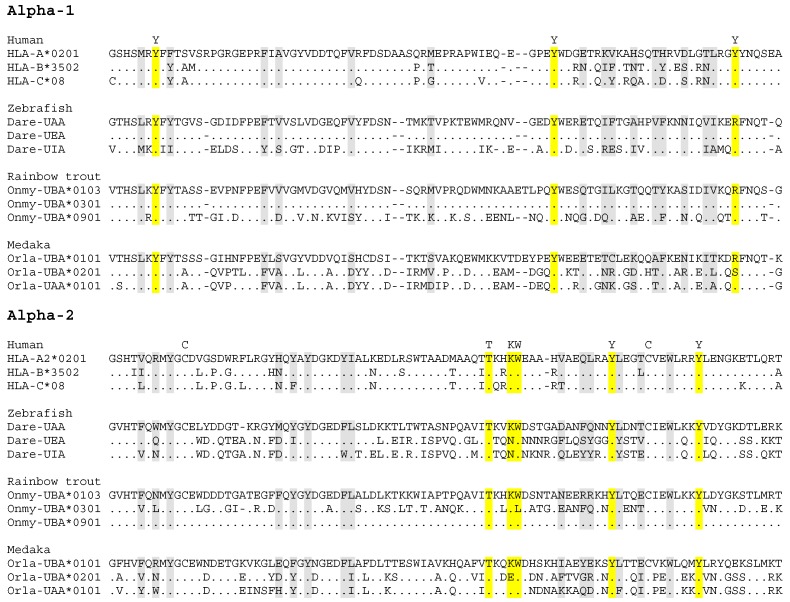
Alignment of deduced classical MHC class I α1 + α2 domain amino acid sequences. This figure is dedicated to showing aspects of within species variation in the most important functional parts of the classical MHC class I molecules, namely the α1 and α2 domains. The three sequences shown each for zebrafish, rainbow trout and medaka were chosen because they reveal past recombination events. In zebrafish, the Dare-UAA and Dare-UEA sequences share an identical α1 sequence, but have very different α2 sequences, indicative of a recombination event involving the intron between the respective exons [11]. On the other hand, Dare-UIA has an α2 sequence which is very similar to Dare-UEA, but has a very different α1 sequence. Among investigated fish species, recombination events involving the intron between the α1 and α2 exons may have been the most abundant in rainbow trout, since many alleles show their recent traces (e.g., [46,113]). This is exemplified here by Onmy-UBA*0103 having an identical versus very different α1 sequence compared to Onmy-UBA*0301 and Onmy-UBA*0901, respectively, whereas the reverse is found for their α2 domains. Investigated medaka haplotypes have two intact classical MHC class I loci, which have consistently been designated *UAA* and *UBA*, although in some haplotypes the “UBA” sequence is quite similar to the UAA sequence (exemplified here by Orla-UBA*0201) and in other haplotypes the UBA sequence is highly divergent from the UAA sequence (exemplified here by Orla-UBA*0101); Nonaka and Nonaka, 2010 [116], explained this situation by interlocus recombination. The levels of divergence among the here depicted zebrafish, rainbow trout and medaka sequences, can be found between individuals of the same species (as allelic or haplotype variation), and are larger than found between three sequences of different human classical MHC class I loci HLA-A, -B and, C. Gray and yellow shading highlight residues that may form part of the peptide binding groove, with yellow shading and a letter indication above the alignment used for conserved residues that bind the peptide ligand termini [41,131]. The letter C above the alignment indicates conserved cysteines. GenBank accessions of the depicted sequences are: HLA-A2, P01892; HLA-B*3502, AAB96790; HLA-C*08, AVQ10002; Dare-UAA, Z46776; Dare-UEA, BC053140; Dare-UIA, KC626502; Onmy-UBA*0103, AF287483 (previous name Onmy-UBA*101); Onmy-UBA*0301, AF287492 (previous name Onmy-UBA*701); Onmy-UBA*0901, AF296366; Orla-UBA*0101, BAD93266; Orla-UBA*0201, AB450999; Orla-UAA*0101, BAD93265.

The first solid study on allelic polymorphism in fish classical MHC class I was performed in sharks [112], showing a similar level of allelic variation as known in mammals [46]. Although unexpected high levels of diversification among classical MHC class I sequences in a single teleost fish species had been reported before [11,46,51], it was only in 2002 that Aoyagi et al., 2002 [113], showed that widely diversified classical MHC class I sequences found in rainbow trout were alleles. For quantitative comparisons of fish and human levels of classical MHC class I variation see the studies by Shum et al., 2001 [46], and McConnell et al., 2016 [54]. The allelic variation in classical class I molecules in intensively investigated teleost fish species extends far beyond the residues that line the peptide binding groove (Figure 2; [43,113,114,115,116]). In a recent article ([117]; see Supplementary File 3 in that article) we summarized the lowest level of amino acid (aa) identity between the deduced α1 + α2 domains (which are the most important domains for function) of reported allelic classical MHC class I sequences within three well-investigated representative teleost species: Zebrafish, 40%; Atlantic salmon, 47%; Medaka, 53%. In comparison, human classical MHC class I molecule HLA-A2 α1 + α2 sequence (GenBank P01892 residues 25–206) can be found to share >80% aa identity with HLA-B and HLA-C sequences (e.g., Genbank AAB96790 and AVQ10002), and shares 75% aa identity with murine H-2K (GenBank AAA39553), and 48% aa identity with a grass carp classical MHC class I sequence (GenBank BAD01658); thus, teleost allelic classical MHC class I variation in the peptide binding domains (which are the domains that interact with T cells) can look like variation between widely divergent species. In investigated Cypriniformes and Salmoniformes the allelic classical MHC class I variation is ancient, with some trans-species lineages for α1 domain sequences inherited from before the separation with eel around 284 MYA [43,46]. In contrast, allelic human classical MHC class I lineages were reported to be only shared with big apes from which the human ancestor only separated ~6 MYA [46,97,118,119]. Variation between the three loci *HLA-A*, *-B* and *-C* can be traced back to the time before the separation between the Apes (Hylobatidae plus Hominidae) and Cercopithecidae (e.g., macaque) ~21 MYA [120,121], but human MHC class I alleles that derived from recombination events that exchanged gene fragments between these loci are rare (e.g., [122]). The most extreme classical MHC class I allelic variation has been described for rainbow trout (*Oncorhynchus mykiss*), with eight highly divergent lineages for the α1 domain, two or three highly divergent lineages for the α2 domain, and even length and sequence variation in the α3 domain, all found in a single gene locus [46,51,80,113,114,123]. The allelic variation in rainbow trout classical MHC class I is further increased by a >10 kb intron between the α1 and α2 domain exons [50], which has been used for allelic recombination events leading to alternative α1-with-α2 combinations (first observed by [46]) as exemplified in Figure 2. The enormous evolutionary pressure necessary to keep this allelic variation appears to be highlighted by the fact that in trout (or other investigated fish) there is no “genomic reservoir” of all these ancient lineages in nonclassical gene or pseudogene copies that could explain the variation in the classical locus by recent interlocus recombination events [43,124]; simply said, the ancient allelic variation appears to be predominantly maintained by selective pressure at the allelic level. Another salmonid fish, Atlantic salmon (*Salmo salar*), also possesses only one polymorphic classical MHC class I gene (named *UBA*) and the allelic variation is reminiscent of that found in trout, with as major difference that most (though not all) of the reported Atlantic salmon sequences have α2 domain sequences of the same lineage [43,114,125,126]. Early research already reported extensive variation among zebrafish classical MHC class I sequences [11,127,128,129], but only in recent years it was realized how this variation is organized at the genomic level [116]. In a corresponding stretch of haploid genome, zebrafish can have either one, two or three classical MHC class I genes, which can be very different from each other and between individuals. Figure 2 shows an example of the variation between zebrafish classical MHC class I sequences encoded by allelic haplotypes, providing evidence of past recombination events involving the intron 2 between the α1 and α2 domain exons. Although in Cypriniformes evidence for such recombination events creating new combinations of α1 and α2 sequences is not as abundant as in Salmoniformes, also in Cypriniformes this type of recombination appears to have been aided by a large size of intron 2 (e.g., [130]). 

In Neoteleostei the evolutionary pressure to maintain ancient classical MHC class I variation appears to be less than in Salmoniformes and Cypriniformes. Namely, in most investigated Neoteleostei only classical MHC class I sequences with α1 domain sequences belonging to only one of the eight α1 lineages found in Salmoniformes (named lineage “α1-I”) were found, and also in the other domains of the classical MHC class I molecules the trans-species lineage variation was not as ancient as found in Salmoniformes [43]. Interestingly, in salmonid evolution the α1-I lineage seems to have been superior to the other α1 domain lineages (lineages α1-II to α1-VIII) for the establishment of new allelic peptide binding groove variation [43], making it an even more fascinating question why species like Salmoniformes kept all those ancient lineages. The best investigated neoteleost species for classical MHC class I variation is medaka (*Oryzias latipes*), with allelic variation determined for two neighboring classical MHC class I genes *Orla-UAA* and *Orla-UBA*, which to some extent experienced interlocus recombination (example shown in Figure 2) and in some alleles also have a relatively large intron between the α1 and α2 domain exons [49,108,115,132]. Although the level of allelic variation is not as ancient and extensive as found in some Cypriniformes and Salmoniformes, and while all medaka classical MHC class I α1 domain sequences belong to lineage α1-I [43], the level of allelic diversification is still impressive and higher and more ancient than found in humans ([108,115] and see the above calculations). It is unclear in how far the medaka classical MHC class I situation is representative for Neoteleostei. Compared to medaka, in the neoteleost fishes stickleback and Atlantic cod the classical MHC class I genes may be characterized by less and younger diversification, whilst having a higher number of classical genes per haploid genome; however, the extent/quality of research in those species probably does not allow final conclusions on the classical MHC class I situation yet [43,117,133,134,135,136]. 

## 5. Functional Analyses of Fish Classical MHC Genes and Molecules

### 5.1. Expression Patterns of Classical MHC Class II

Analyses with Northern dot blots, RT-PCR or polyclonal antisera showed that classical MHC class II genes/molecules in teleost fish are predominantly expressed in B-lymphocytes (e.g., [58,59]) and in polymorphic cells presumably involved in antigen presentation in the thymus [60] and other tissues [61,62]. This expression pattern resembles that of mammalian MHC class II. Furthermore, teleost classical MHC class II expression can be upregulated after immune stimulation (e.g., [63,64,96]), which also is reminiscent of the situation in mammals and which agrees with conserved promoter elements [64,65].

### 5.2. Expression Patterns of Classical MHC Class I

Northern blot data indicated that classical MHC class I in shark (e.g., [137]) and teleosts (e.g., [80]) are expressed ubiquitously, with highest expression in lymphoid and epithelial tissues. In rainbow trout this was confirmed at the cellular level by an established monoclonal antibody, showing that classical MHC class I molecules were predominantly found in epithelial cells, endothelial cells, and leukocytes [81], as in mammals. By using an established polyclonal antiserum, a similar expression profile was found for classical MHC class I in stickleback [62]. Various studies showed that fish classical MHC class I gene expression can be enhanced after immune stimulation (e.g., [82,85,138,139]), in agreement with conserved promoter elements [50,82,140,141]. Notable is that in common carp the cell-surface expression levels of classical MHC class I were substantially less at lower temperatures, consistent with the lower amounts of β_2_-m transcripts found at those temperatures [83]. It has to be realized that fish are ectotherm species, and that the fish adaptive immune system does not work equally well under each naturally encountered temperature [142,143].

### 5.3. Binding of Peptide Ligands by Classical MHC Class I Molecules

In mammals, both classical MHC class I and MHC class II bind peptide ligands in the groove formed by their membrane-distal domains, but whereas for teleost fish classical MHC class I this has been confirmed, for fish MHC class II this has not been investigated yet. Classical MHC class I heavy chains form complexes with a single immunoglobulin-like domain molecule beta-2-microglobulin (β_2_-m), which was also found to be the case in teleosts [84,85,86]. The binding of β_2_-m to the heavy chain is unstable, unless simultaneously a peptide of ~9 aa binds into the groove of the heavy chain (synergistic heterotrimer complex formation), as was also found for teleost fish [85,86]. A recent milestone in fish MHC research was the elucidation of a grass carp heavy chain/β_2_-m/peptide heterotrimer structure, which was found to be similar to such structures in tetrapod species [86]. Recently, also interaction was shown between rainbow trout classical MHC class I and tapasin [144], providing additional evidence for similarities in the peptide presenting functions between teleost fish and mammals. 

### 5.4. MHC class I Restriction of Cell-Mediated Cytotoxicity by Lymphocytes

Specific cell-mediated cytotoxicity by lymphocytes in ginbuna crucian carp was found to require syngeneity between the effector cell donor and the target cells [89,90,91,92], and in mammals such genetic restriction involves classical MHC class I. Linkage association studies suggested the need of matching classical MHC class I markers for specific cell-mediated cytotoxicity in rainbow trout, but the experimental setups have been too limited to allow firm conclusions on MHC restriction [93,94]. In grouper, a neoteleost fish, direct evidence was provided of specific cell-mediated cytotoxicity against virus-infected autologous cells by CD8^+^ lymphocytes [95]. In summary, there probably is MHC restriction in fish, but final evidence remains needed.

### 5.5. Additional, Indirect Indications for Classical MHC Functions in Fish

Except for the above-mentioned, there are also (other) indirect indications for MHC functions in fish similar to as in mammals. For example, clonal expansions of systemic TCRαβ T cells upon specific immune stimulation have been observed, and there are many observations that support the existence of similar cytokine networks and helper and regulatory T cell functions (e.g., [68,69]; reviewed in [70]). Furthermore, also the T cell education system, concerning the tissue organization of the thymus and the existence of CD4^−^CD8^−^, CD4^+^CD8^+^, CD4^+^CD8^−^ and CD4^+^CD8^−^ thymocytes, seems to be similar between teleost fish and mammals [60,71,72,73,74]; the shark thymus may be similarly organized, but has been studied less intensively [145]. Teleost fish classical MHC class II molecules possess conserved residues that in mammals can bind to the TCR co-receptor molecule CD4 on helper/regulatory T cells [27] and teleost fish classical MHC class I molecules possess conserved features that in mammals are involved in binding the TCR co-receptor CD8 on cytotoxic T cells [41,113,146]. The CD8 molecule on mammalian cytotoxic T cells is a heterodimer of an alpha and a beta chain, CD8α and CD8β, and fish have genes for both components [147,148,149,150,151]; the cytoplasmic tail of teleost fish CD8α was shown to have LCK kinase binding properties as known in mammals [152]. Unlike mammals, teleost fish have two CD4 molecules, CD4-1 and CD4-2, which mostly are co-expressed by the same T cells and which both have cytoplasmic tails that can bind LCK kinase as known for mammalian CD4 [74,75,76,77,78]; the reason for this is not known. Like mammals, teleost fish also have LAG-3 as a potential receptor molecule for MHC class II complexes [77], but the function of fish LAG-3 has not been studied yet. Similar pathways in fish and mammals for MHC intracellular transport and loading with peptide ligands is suggested by fish possessing a similar set of specialized molecules as known in mammals, such as PSMB, TAP and tapasin molecules for the MHC class I system (see below), and CD74 (aka invariant chain or Ii; [67,79,153]) for the MHC class II system. Peculiarly, teleost fish have two CD74 molecules, CD74a and CD74b, the function of which is not known [59,67,79,154]. A strong indicator for a similar MHC class II functional system as in mammals seems to be that in teleost fish that lost their MHC class II genes also the *CD4-1*, *CD4-2*, *LAG-3*, *CD74a* and *CD74b* genes tend to be lost or to have lost their original function [31,32,117,155].

## 6. Genomic Organization/Haplotype Variation

In teleost fish, classical MHC class I and II are not linked, and only classical MHC class I genes reside in typical *Mhc* genomic regions. Throughout jawed vertebrate species, despite individual differences, typical *Mhc* genomic regions are found with classical MHC genes plus a conserved set of non-MHC genes amongst which genes for proteins involved in the classical MHC class I peptide loading pathway (reviewed in [156]). After fragmentary reports (e.g., [11,51,55]), Clark et al., 2001 [52], were the first to report a consecutive sequence of a teleost Mhc genomic region (in Fugu). Currently, *Mhc* genomic regions have been analyzed for a considerable number of teleost fish species (e.g., [27,43,50,53]). Distribution in different genomic linkage groups (nonlinkage) of classical MHC class I versus classical MHC class II in the teleost genomes was first reported for zebrafish by Bingulac-Popovic et al., 1997 [47], and later confirmed for other teleost fishes (e.g., [27,48]). Pipefish and, independently, Gadiformes including Atlantic cod (for phylogeny see Figure 1) apparently even lost MHC class II function [31,32,33,117]. Meanwhile, shark classical MHC class I and II genes were found to be conventionally linked in a typical *Mhc* region as known in tetrapods, concluding that such linkage is the ancestral situation [157,158,159]. Data suggest that in primitive ray-finned fish, like spotted gar, classical MHC class I and II genes may still be linked together, whereas in teleost fish only the classical MHC class I genes remained in a typical *Mhc* region and the classical MHC class II genes were translocated to other chromosomes [27]. The whole genome duplication early in the teleost lineage resulted in duplications of the *Mhc* region, but one of the duplicated regions, which lost all classical MHC genes and only in some teleost species retained nonclassical MHC class II [27], is usually not discussed as an *Mhc* region and will be neglected in the remaining of this article.

### 6.1. Teleost Fish Mhc Allelic/Haplotype Sequence Variation in PSMB and TAP2 Genes

At least in mammals, a large cytoplasmic protein complex with peptidase properties called the “proteasome” generates peptides that can be transported through the membrane of the endoplasmic reticulum by heterodimer transporters associated with antigen processing 1 and 2 (TAP1 and TAP2; aka ABCB2 and ABCB3) complexes, and then can be aided/modified/selected by a number of molecules including tapasin (aka TAP binding protein or TAPBP) to bind in the classical MHC class I peptide binding groove [14,160]. During infection, at least in mammals, the proteasome beta (PSMB) subunits PSMB5, PSMB6 and PSMB7 are (partially) exchanged by PSMB8 (aka LMP7), PSMB9 (aka LMP2), PSMB10 (aka MECL1), respectively, creating an “immunoproteasome” with different protease properties, which leads to the generation of different peptides that are presented by classical MHC class I [14,161]. In teleost fish *Mhc* regions, as is probably inherited from a jawed vertebrate ancestor, a gene organization similar (though not identical) to that in many other jawed vertebrates can be found, namely with classical MHC class I genes linked with *PSMB8*, *PSMB9*, *PSMB12*, *PSMB13*, *TAP2* and *tapasin* genes, and in some fish species with *PSMB10* (reviews [56,156]). PSMB12 (previously also named LMP2-like, LMP2/δ, PSMB9L or PSMB9B) and PSMB13 (previously also named PSMB7 or PSMB10) genes are members of the PSMB6/9/12 and PSMB7/10/13 families, respectively, found in teleost fish, and their levels of divergence from the respective other two family members suggest that they may be ancient [51,54,56,57]. The implication of these teleost genes in immune responses was not only suggested by their linkage within the *Mhc* region, but also by the increased expression of teleost *PSMB8*, *PSMB9*, *PSMB10* (named *PSMB7* in [162]), *PSMB12*, *PSMB13*, *TAP2* and *tapasin* after immune stimulation (e.g., [162,163,164,165]). In humans, the molecules involved in the classical MHC class I peptide loading pathway show little allelic variation (e.g., [54]), but in rat allelic variations in TAP2 are found which appear to affect the peptides that can be presented by classical MHC class I [166,167,168] and a similar situation is found for TAP1, TAP2 and tapasin in chicken [169,170,171]. In the frog Xenopus, not only ancient allelic classical MHC class I variation is observed, but also highly diverged allelic forms of PSMB8, TAP1 and TAP2, and some variation in PSMB9 [54,172,173]. In several investigated teleost fish, like medaka, rainbow trout, Atlantic salmon and zebrafish, very divergent and ancient variation is observed for PSMB8, which is represented by two lineages called PSMB8A and PSMB8F [49,54,56,174]. The PSMB8A and PSMB8F lineages were already established at the level of cartilaginous fish, their sequences can show >30% amino acid sequence divergence from each other, and they are predicted to confer different protease properties to the immunoproteasome [174,175,176]; although not necessarily at identical location within the different *Mhc* haplotypes, it was found that *PSMB8A* and *PSMB8F* sequences can segregate in a functionally allelic manner in zebrafish and medaka [176]. Based on inconclusive experiments it was prematurely hypothesized that also in rainbow trout full-length *PSMBA* and *PSMBF* genes segregate as functional alleles [176], whereas there is only evidence for intact trout *PSMB8A* and *PSMB8F* genes being located on different chromosomes without indications for significant allelic variation (see the paragraph below and [56]). Considerable allelic/haplotype sequence divergence can, in some teleost fish species, also be found for *Mhc*-situated *PSMB9*, *PSMB13* and *TAP2*, and zebrafish allelic/haplotype variation for their encoded products can be as high as 14%, 29% and 50% amino acid divergence, respectively ([49,56,176]; calculations by [54]). Zebrafish may also have *Mhc* haplotypes without *PSMB12* (null-allele variation) [54]. In the *Mhc* regions of Salmoniformes, notable levels of polymorphism in the peptide loading pathway genes could not be found, with the exception of *TAP2* [50,56].

The analyses of *Mhc*-situated *PSMB*/*TAP*/*tapasin* genes have not been sufficiently exhaustive yet for allowing a definite comparison between teleost fishes on levels of within species allelic/haplotype divergence. In summary, in some teleost fish species, classical MHC class I genes displaying unprecedented levels of allelic/haplotype divergence are closely linked with peptide loading pathway genes that also display considerable allelic/haplotype sequence divergence. Whether the latter has a function in providing the most suitable peptides for binding the classical MHC class I molecules encoded by the respective *Mhc* haplotype, or mainly functions to further increase variation in peptide/MHC complexes between individuals of the same species, remains to be determined.

### 6.2. Copy Number Differences in MHC Class II Genes

Although in humans the copy number of MHC class I and II genes does not largely vary between individuals, more notable differences in MHC gene numbers between individuals were reported for various other species such as for example rat [177], quail [178], and the frog Xenopus [179]. Also in shark (e.g., [156,180]) and teleost fish, differences in MHC gene number can be observed. Extensive MHC class II B copy number variation in cichlid fishes was concluded [181] and copy number variation was also shown for MHC class II A in the cichlid tilapia [182]. For a summary of observed or suggested MHC class II gene copy number variation in several teleost fishes see Text S3 in reference [27].

### 6.3. Copy Number Differences in MHC Class I Genes

Southern blot data indicated that rainbow trout individuals differ in their genomic copy number for the MHC class I lineages U, S and L (e.g., [45,106]). Although variation in Z gene sequences between rainbow trout individuals was reported at the cDNA level [124], allelic or copy number variation in salmonid Z genes has not properly been investigated. As mentioned above, zebrafish has a variable number of classical U lineage genes in its *Mhc* region, but it also has a nonclassical U lineage gene situated on another chromosome which displays null-allele (presence/absence of intact gene) variation [183]. Data suggest that zebrafish have copy number variation in genes of the nonclassical MHC class I lineages L and Z, but genes of these lineages are not linked to the zebrafish *Mhc* [105,107]. Some of the zebrafish L lineage genes, however, can be found linked to the classical MHC class II genes [45]. In Neoteleostei, in cichlid fishes and Atlantic cod data suggested copy number variation in U lineage genes [133,134,184,185], and in medaka probable null-allele variation was found for a nonclassical gene of the U lineage situated within the *Mhc* region [49]. In summary, copy number variations in both MHC class I and II genes appear to be common in fish. Null-allele variation in nonclassical MHC class I genes is particularly interesting because in mammals it is known that knockout of such genes can deplete T cell subpopulations that are restricted by their products [186], like for example *MR1* and *CD1d* knockout cause depletions of MAIT cells [187] and NKT cells [188], respectively.

## 7. The Genomic Organization of the Classical MHC Gene Loci and the Duplicated *Mhc* Regions *Onmy-IA* and *Onmy-IB* in Rainbow Trout

**Figure 3:**
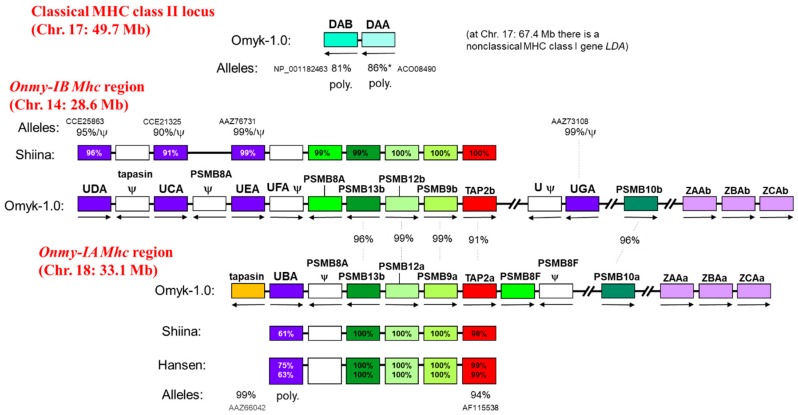
Schematic organization of the rainbow trout *Onmy-IA, Onmy-IB* and classical MHC class II loci. MHC, PSMB, TAP2 and tapasin genes are indicated by blocks, and double slashes indicate that there is a short stretch with other genes between them (for those genes see [56]). Blocks representing intact genes are colored based on identity or molecule family, and white blocks indicate pseudogenes (ψ for pseudogene). The indicated chromosome positions are based on the rainbow trout whole-genome dataset Omyk_1.0 accessible at NCBI (https://www.ncbi.nlm.nih.gov/assembly/GCF_002163495.1/). For the *Onmy-IA* and *Onmy-IB* regions, not only the gene organizations found in Omyk_1.0 are shown, but also the shorter stretches reported by Shiina et al., 2005 [50], and Hansen (GenBank HM210571). Numbers indicated within the blocks for the Shiina genes refer to the amino acid identity percentages when comparing with the products of the matching Omyk_1.0 gene. This is done similarly for the Hansen genes, with the first number based on comparison with the matching Omyk_1.0 gene, and the second number based on comparison with the matching Shiina gene. For the PSMB and TAP2 genes, the amino acid identities between the Omyk_1.0 *Onmy-IA* and *Onmy-IB* encoded gene products are also given (the numbers on top of dashed lines). The only classical MHC class I gene is the *UBA* gene situated in the *Onmy-IA* region, and the only classical MHC class II genes are *DAA* (encoding an alpha chain) and *DAB* (encoding a beta chain). They are known to be polymorphic (poly., see the main text). As an approximate measure for levels of variation, if deemed possible and interesting, in the “Alleles” sections for many genes the maximum divergent allelic molecules compared with the Omyk_1.0 encoded gene products are listed with their GenBank accession numbers and the percentages of amino acid identity that they share with the Omyk_1.0 encoded molecules; if in addition for these genes also indications for null-alleles were found (see main text), that is shown by ψ symbol. Arrows indicate gene orientations.

Salmonid fishes probably experienced an additional whole genome duplication event around 60-90 MYA (SGD for salmonid-specific whole genome duplication; [50,189,190]). Figure 3 schematically shows the organization of (i) the rainbow trout *Mhc* region harboring a polymorphic classical MHC class I locus on Chr. 18 (aka linkage group LG-16 or RT-16) which has been called the *Onmy-IA* region, (ii) its SGD-derived duplicated *Mhc* region without classical genes on Chr. 14 (aka LG-3 or RT-3) which has been called the *Onmy-IB* region [50], and (iii) the polymorphic MHC class II locus on Chr. 17 (aka LG-29 or RT-29). Whereas for the single classical MHC class I gene, *UBA* on Chr. 18, extreme levels of allelic diversification were observed (see above; [43,113,114]), this was not the case for four nonclassical genes of the U lineage on Chr. 14, named *UCA*, *UDA*, *UEA* and *UGA* [114,124,191]. Whereas *UCA* and *UDA* are quite similar to each other and even recombined with each other [191], all these nonclassical sequences are considerably different (showing <70% aa identity over the encoded full-length sequence) from the classical *UBA* sequences, and *UEA* and *UGA* are considerably different from each other and from *UCA*/*UDA* [50,124]. The *UCA* and *UDA* genes display some degree of allelic variation, which unlike in classical sequences is not predominantly dedicated to the membrane-distal domains, while *UEA* and *UGA* seem to be close to monomorphic [124,191]. However, importantly, for *UCA* and *UDA* [124,191], as well for *UEA* [124] and *UGA* ([124]; our unpublished results), also presumable null-allele (pseudogene) sequences or indications for null-alleles were found. The rainbow trout *UCA*, *UDA*, *UEA* and *UGA* genes may all be involved in the immune system, as suggested by their enhanced transcription after viral infection [192]. Among these nonclassical MHC class I molecules, only UGA may bind peptides in a manner very reminiscent of classical MHC class I [124], but in rainbow trout the *UGA* 5′-UTR has additional AUGs plus an inverted repeat (suggesting regulation at the translation level; GenBank accession EU036647) and the UGA cytoplasmic tail has a typical dileucine endosomal targeting motif (GenBank accession AY253140; [193]), which together with the apparent lack of polymorphism argues against classical character. As already concluded previously for Salmoniformes [56], there are no indications for important allelic/haplotype variations for rainbow trout PSMB and tapasin genes situated in the *Mhc* region, but for *TAP2a* (the small font letter “a” refers to being situated in the *Onmy-IA* region) there are indications for 6% allelic amino acid divergence (Figure 3; [50,51]). The investigated *Onmy-IA* and *Onmy-IB* haplotypes each contain three genes of the nonclassical MHC class I lineage Z [56], but there is no information about their possible allelic/haplotype variation.

As mentioned above, rainbow trout have only one classical MHC class II locus with one alpha gene and one beta gene, both which are polymorphic, situated on Chr. 17 (Figure 3). On the same chromosome, at a far distance of ~18 Mb, a gene of the nonclassical MHC class I lineage L, *LDA* [45], is situated, but there is no information suggesting important polymorphism or null-allele variants of that locus.

In short, (1) *Onmy-IA* haplotypes display extreme sequence divergence in the single classical MHC class I gene *UBA* (see the paragraph on allelic polymorphism) and some sequence variation in the associated *TAP2a* genes, (2) the most dramatic variation observed among the *Onmy-IB* haplotypes probably concerns the possible null-allele variation for the nonclassical genes *UCA*, *UDA*, *UEA* and *UGA*, and (3) the trout classical MHC class II locus is characterized by polymorphism of both the single alpha and single beta genes.

In Atlantic salmon, the organization of the *IA*, *IB* and classical MHC class II loci is quite similar, though not identical, to the one shown for rainbow trout in Figure 3 [56,104,194].

In Figure 3, only the MHC loci in rainbow trout are shown for which extensive variation is well documented. Also on other trout chromosomes MHC genes are located, but they do not overlap with the confidence intervals of the most interesting reported QTL, and will not further be discussed in this article: Chr. 2, nc II (nonclassical MHC class II); Chr. 3, nc II; Chr. 6, nc I (L lineage); Chr. 12, nc II; Chr. 13, nc II; Chr. 22, nc I (U and L lineages); Chr. 24, nc I (S lineage), Chr. 26, nc I (L lineage).

## 8. Association of Teleost Fish MHC Genes with Disease Resistance

Rainbow trout and Atlantic salmon are the only fish species for which disease resistance related genome-wide QTL studies can readily be linked with whole genome sequence information [190,195] available at the chromosome and linkage group levels (e.g., [196,197]), and for which the locations of the classical MHC class I and II loci are known; for rainbow trout, the *Onmy-IA*, *Onmy-IB* and classical MHC class II loci (see Figure 3) were also physically mapped to chromosomes 18, 14 and 17, respectively [50,198,199]. In Atlantic salmon, the corresponding *Sasa-IA*, *Sasa-IB* and classical MHC class II loci were mapped to chromosomes 27, 14 and 12 in that species [194]. Table 2 summarizes the relevant QTL studies, with underlining highlighting the most important QTL in the respective study. In both rainbow trout and Atlantic salmon, the chromosomes with the *IB* region and the classical MHC class II locus were found among the linkage groups with suggestive or significant QTL. Ozaki et al., 2001 and 2007 [200,201], found that the major QTL in rainbow trout for resistance against IPN virus mapped to a large part of Chr. 14 that includes the *Onmy-IB* region, but finer mapping was not performed. Palti et al., 2015 [202], found a QTL on trout Chr. 14 for resistance against the bacterium *Flavobacterium psychrophilum* (cold water disease), but the most likely region on Chr. 14 for that QTL does not include the *Onmy-IB* region. Moen et al., 2009 [203], mapped a suggestive QTL for resistance against IPN virus in Atlantic salmon to a large region (the region upstream of negative marker BHMS429) of Chr. 14 where the *IB* region in that species (the *Sasa-IB* region) is located, but fine mapping was not performed. Gonen et al., 2015 [204], mapped a suggestive QTL for resistance against SAV virus in Atlantic salmon to Chr. 14, but from their study we are unable to understand the location of the confidence interval of that QTL on the chromosome. Ozaki et al., 2007 [201], mapped a suggestive QTL for resistance against IPN virus in rainbow trout Chr. 17, with the region of most likelihood laying not far from (but not including) the classical MHC class II locus. Khoo et al., 2005 [205], mapped a single QTL for resistance against IHNV virus in rainbow trout to a large region (for location compare [205] with [201]) that includes the classical MHC class II locus, but fine mapping was not performed. In the study by Fraslin et al., 2018 [206], a consistent QTL for resistance against cold water disease in rainbow trout was found on Chr. 17, but the most likely region for that QTL does not include the classical MHC class II locus (see their Table S6 [206]). Verrier et al., 2013 [207], mapped a QTL for resistance against VHS virus in trout to a part of Chr. 17, which does not include the classical MHC class II locus (compare with their Table S1 [207]). In summary, disease resistance related QTL studies in rainbow trout and Atlantic salmon suggest a possible QTL effect of the *IB* and classical MHC class II loci, but much finer mapping is necessary to substantiate those hypotheses. Importantly, however, the salmonid *IA* regions with the classical MHC class I genes are not associated with any of the reported QTL.

In other fish species, there also have been a number of genome-wide QTL studies on disease resistance traits, but more information is necessary to interpret them in regard to MHC genes. However, although the location of the classical MHC class I genes is not known in that study, it is interesting that Sawayama et al., 2017 [219], mapped a suggestive QTL for resistance against red sea bream iridoviral disease (RSIVD) in red sea bream to a relatively small confidence interval region that includes classical MHC class II (but finer mapping remains necessary).

Notable are also two studies in Salmoniformes that compared possible effects on differences in disease resistance by the *IA*, *IB* and classical MHC class II linkage groups. Miller et al., 2004 [220], found differences in resistance against IHN virus in Atlantic salmon to be linked with the *Sasa-IB* region, and maybe with the classical MHC class II locus, but not with the *Sasa-IA* region. Johnson et al., 2008 [221], found differences in resistance against the bacterium *F. psychrophilum* in rainbow trout to be linked with *Onmy-IB* and the classical MHC class II locus but not with *Onmy-IA*. Thus, also those two studies suggest that the enormous allelic divergence in the classical MHC class I genes in the *IA* regions has no notable impact on disease resistance.

## 9. Association of Teleost Fish MHC Genes with Allograft Rejection

In teleosts, both the classical MHC class I and classical MHC class II linkage groups have been associated with allograft rejection. Namely, linkage association studies indicated that MHC class II in gila topminnow is an important marker for scale allograft rejection [66] and that classical MHC class I in rainbow trout is an important marker for erythrocyte allograft rejection ([87] plus http://dx.doi.org/10.1007/s00251-003-0632-3, which the journal originally forgot to print). The erythrocyte allograft rejection in rainbow trout needed previous sensitization or took several weeks and was coincident with CD8 upregulation, thus suggesting T-cell involvement [87,222]. In contrast, studies in channel catfish found association of the classical MHC class I linkage group with immediate spontaneous cell-mediated cytotoxicity against allogeneic cell lines, which is reminiscent of NK-cell activity [88]. The relative importance of NK-cell and T-cell activity in MHC-dependent allograft rejection in fish remains to be determined.

In recent years, it has become accepted that, although rare, mammals can transmit cancer cells by contact over mucosal tissue, as exemplified by a facial tumor in Tasmanian devil and a venereal cancer in dogs [223,224]. Furthermore, spread of transmissible cancer cells among mollusks suggest that these cells can survive transport through water [225]. Because teleost fish live in water and are fully covered by mucosal tissue (they have “living skin” without an outer layer of dead epithelial cells), and, dependent on the species, can live in groups and can be cannibalistic, their intensity of grafting cancer cells and other cells to each other may be more intensive than in mammals. Since allografting has the potential risk of inducing cancer (see above) or graft versus host reaction (GVHR; [226]), teleost fish may need an enhanced ability to kill allografted cells, and we speculate that this at least in part can explain their MHC situation. Namely, the nonlinkage of classical MHC class I and II is expected to enhance the allogeneic variety among the individuals within teleost populations, and the wide allelic/haplotype divergence of the classical MHC class I sequences may provoke a strong immune response by cytotoxic T cells and/or NK cells. Because allelic classical MHC class I molecules in teleosts can have divergence levels which in mammals are only known among xenografts (see above), we speculate that teleost NK cells may play an important role in allograft rejection (for NK cells and xenografts see the review by [227]).

## 10. Association of Teleost Fish MHC Genes with Partner Selection

There is a line of research which claims an association between MHC variation and preferences for sexual or non-sexual partners in a wide variety of animals including teleost (e.g., reviewed in [228,229]). However, the claims for such associations may not be fully convincing and have been debated (e.g., [230,231]). The most prominent study claiming an MHC-based selection of sexual partners in fish was in stickleback and had Reusch et al., 2001 [232] conclude that female sticklebacks choose their mating partners by MHC class II B gene “counting” in order to assure optimal gene copy numbers in their offspring. However, in our opinion, the setup of this study [232] was lacking in scientific quality, as it was essentially based on comparing the number of MHC class II B fragments amplified by PCR from genomic DNA. Namely, (1) it was not investigated whether the amplified genomic fragments represented intact genes or pseudogenes, (2) it was not determined whether the primers used were exhaustive in amplifying all stickleback MHC class II B alleles present in the investigated population, and (3) the statistics suffered from a lack of prediction. A recent QTL study that investigated partner selection by female sticklebacks only found significant markers on Chr. XIV and Chr. XXI [233], whereas the classical MHC class II loci of stickleback probably are located on Chr. VII [27,234].

Overall, as probably follows from our organization of this paragraph, we are quite skeptical about the research field specifically dedicated to the correlation of MHC with partner choice. 

## 11. Association of Teleost Fish Classical MHC Class I with Behavior and Behavior-Related Growth

For a long time, neurons were thought to be immunoprivileged cells without classical MHC class I expression. However, Corriveau et al. found in 1998 [235] that classical MHC class I in mammals is expressed in some neurons during neuron rearrangement. The authors naturally suggested that the molecules might have a function in such rearrangement. Later studies showed that *β_2_-m* knockout mice, which should be deficient in most MHC class I cell surface expression, have aberrant phenotypes in brain morphology [236] and sexual behavior [237]. Huh et al., 2000 [236], found that in these mice synaptic connections were stabilized by an increase of long-term potentiation and the lack of long-term depression, and Oliveira et al., 2004 [238], found an increased reduction of their synapses after transection of spinal motor neurons. Mice lacking classical MHC class I molecules (KbDb-knockout) have less synapse elimination compared with wild type (WT), and elimination can be restored to WT levels by selectively expressing H2-Db in LGN neurons [239]. Furthermore, pyramidal neurons in KbDb-knockout mice have more extensive cortical connectivity than normal [240]. However, the molecular cascade by which classical MHC class I molecules affect neural plasticity probably is still not understood [240,241,242]. To our knowledge, the effect of allelic MHC variation on mammalian brain or neurons has not been investigated yet, although in humans a linkage association was found between the *Mhc* region and schizophrenia (reviewed in [243]).

Using a monoclonal antibody established against rainbow trout classical MHC class I, we found staining in some neurons in the brain stem of early rainbow trout fry [60]. We speculated that if classical MHC class I has a function in neural arrangement, as found for mammals, the enormous allelic variation between rainbow trout individuals could lead to differences in their neural systems. A trait influenced by brain development and under balancing selection is behavior [244,245,246]. We therefore studied the linkage association of rainbow trout classical MHC class I (locus *UBA* in the *Onmy-IA* region) with boldness/aggressiveness versus carefulness/friendliness, because these behavior traits are relatively easy to study as a dimorphism. In a series of experiments independently performed in two different institutes we demonstrated that within the investigated strain of rainbow trout the UBA*401 allele marker was associated with bold/aggressive behavior and fast growth, whereas the UBA*4901 allele marker was associated with careful/friendly behavior and comparatively slow growth [247]. The data suggested that the growth differences were caused by differences in feeding and swimming behavior during social competition [247]. However, although we had our initial data confirmed in another institute and the statistics looked convincing [247], currently we are somewhat skeptical about our model because in the bulk of QTL studies on fish behavior a possible linkage between the classical MHC class I locus and behavior was not found (e.g., [248,249]). The only genome-wide QTL report that might support our finding is a study in tilapia that reported linkage association between the classical MHC class I locus and stress-related features [250].

In the future, to obtain final evidence of whether classical MHC class I allelic/haplotype differences can cause differences in the fish nervous system and behavior, it probably will be best to start using transgenic zebrafish or medaka.

## 12. Discussion

In disease resistance QTL studies in rainbow trout and Atlantic salmon, linkage groups with the highly polymorphic classical MHC class I locus (the *IA* region) were not among the reported QTL regions, whereas QTL were found to be linked with a region including several nonclassical MHC class I genes (the *IB* region) and the region including the classical MHC class II locus (see also [201,251]). Fine mapping and analysis of the respective MHC alleles remains necessary. In case the classical MHC class II locus would be directly responsible for the observed QTL, we speculate that the probable cause concerns allelic differences in peptide binding grooves and the consequential presentation of different peptides, whereas if the *IB* region would be directly responsible, we assume that the probable cause more likely concerns the presence versus absence of certain nonclassical MHC class I genes (null-allele variation) and the consequential absence/presence of T cell subpopulations (although in fish such correlation remains to be determined). We have no hypothesis for why peptide groove variation in MHC class II might cause bigger differences in disease resistance than peptide groove variation in MHC class I. Modern techniques for making transgenic fish, combined with the large number of offspring in single broods of some species like Salmoniformes, would probably allow a final assessment of the theory that different MHC alleles can cause differences in disease resistance against pathogens. Currently, that popular theory is debatable to some extent—even for mammals.

It is puzzling why the allelic classical MHC class I variation in many teleost fish is so divergent and ancient. The maintenance of these ancient allelic lineages probably can’t be explained by differences in peptide binding properties, because only some and not all ancient lineages are characterized by unique properties of the peptide binding groove [43] and the variable positions extend far beyond the peptide binding groove (e.g., Figure 2). Especially allelic length and sequence variation in the α3 domain [113] is difficult to explain with a model solely based on pathogen-driven selection for presenting different peptides. The most straightforward hypothesis to explain this extreme classical MHC class I allelic divergence is that it was selected to enhance the vigor of allograft rejection, because that is the process most readily observed in association with allelic MHC variation, and that is the process from which the MHC molecules derived their name. However, for the moment that is only speculation, and more research is needed on MHC-induced allograft rejection in fish.

Classical MHC molecules present a wide variety of peptides, and most pathogens may provide enough different antigens for potentially inducing adaptive T cell responses. Naturally, if immune responses based on subunit or peptide vaccines would be measured, the chances of finding important effects of MHC allelic variation should be higher. The QTL studies listed in this study concerned primary challenges with pathogens, and it would be interesting to perform some QTL studies in fish that would be more dependent on immune memory.

As shown in Figure 3, the MHC situation in rainbow trout provides a unique situation with very different types of allelic/haplotype variation in unlinked classical MHC class I, classical MHC class II, and a fragment with several nonclassical MHC class I. We hope that in the future, more intensive investigation of the influences of these variations on the trout immune system (e.g., on the selected NK and T cell populations) may benefit both fish aquaculture and the general understanding of MHC evolution.

In conclusion, the impressive MHC polymorphism originally found in mice and humans is a common trait among jawed vertebrates including fish. This indicates important evolutionary advantages that likely involve the resistance against pathogens as one of the relevant phenotypes. However, despite decades of knowledge of MHC polymorphism in an increasing number of model species, conclusive evidence for any of the elegant explanatory theories has not been obtained. Classical MHC class I molecules in many teleost fish show allelic variation at two distinct levels, namely the level of relatively young peptide binding groove variation as known in mammals (within lineage variation) and the level of much older variation that also concerns residues outside the binding groove (between lineage variation). For both levels, a strong signature of evolutionary selection is observed. Those two different levels of allelic variation may be driven by different functions, possibly not only involving pathogen resistance. This situation in fish might be fundamentally different from the situation in mammals, but alternatively might represent a more extreme case of a situation that has not yet been recognized in mammals due to its subtlety. Possibly, knowledge of fish MHC may contribute to a better understanding of mammalian MHC.

## Figures and Tables

**Table 1:** Summary of MHC (system) traits in teleost fish compared to mammals. *(important references are between brackets).*

**MHC molecule types** ***MHC class I versus class II*** Like mammals, most fish possess MHC class I, MHC class IIα, MHC class IIβ and β_2_-m molecules. However, some teleost fish lost MHC class II genes in their evolution. [24,31,32,33,34] ***Classical versus nonclassical MHC class II*** As in mammals, in fish both polymorphic classical and nonpolymorphic nonclassical MHC class II are found. The nonclassical MHC class II lineages in teleost fish are not the same as in tetrapods, and, unlike the DM lineage distribution in tetrapods, they are not stably inherited throughout those teleosts that do possess an MHC class II system. [27,35,36,37,38,39] ***Classical versus nonclassical MHC class I*** Like mammals, all investigated teleost fish possess at least one polymorphic classical MHC class I gene and a number of nonpolymorphic nonclassical ones. The conclassical MHC class I lineages found in tetrapods and fish are not the same. Different from mammals is that throughout ray-finned fish members of an ancient nonclassical MHC class I lineage named "Z" are conserved which show highly conserved features for presumably binding a (modified) peptide ligand, while the Z lineage was lost in tetrapods. [24,40,41,42,43,44,45,46] **Genomic organization** ***Genomic location of classical MHC class II genes*** Unlike in mammals, teleost fish classical MHC class II genes are not situated in typical *Mhc* regions and are not linked with the classical MHC class I genes. Even between teleost fish species differences in genomic locations of the classical MHC class II genes can be observed, highlighting the relative plasticity of the teleost fish MHC class II system. [27,47,48] ***Genomic location of classical MHC class I genes*** As in mammals, fish classical MHC class I genes are linked together with TAP, tapasin, PSMB and other conserved "framework" genes in a typical *Mhc* region. [11,49,50,51,52,53] ***PSMB, TAP and tapasin genes in the Mhc region*** As in mammals, PSMB, TAP and tapasin genes are located in the teleost fish *Mhc* region, but their number and lineages differ. Unlike in mammals, teleost fish *TAP1* is not linked with the *Mhc* region. Compared to mammals, teleost fish have additional ancient gene copies of the PSMB6/9/12 and PSMB7/10/13 lineages. In some but not all teleost fish, different from humans, considerable allelic or haplotype variation can be found for the *Mhc*-situated PSMB and TAP2 genes. [54,55,56,57] **Functions of classical MHC class II** ***Expression of classical MHC class II*** As in mammals, teleost fish classical MHC class II transcripts and molecules appear to be expressed in professional antigen presenting cells like for example B lymphocytes. Furthermore, as in mammals, their expression is upregulated after immune stimulation, which agrees with conservation of promoter motifs similar to those in mammals. [58,59,60,61,62,63,64,65] ***Peptide presentation by classical MHC class II*** For fish MHC class II, the structure or functions in peptide presentation have not been determined yet. However, fish classical MHC class II molecules have a set of conserved residues which suggest a similar mode of peptide ligand binding as known in mammals. [27] ***Allograft rejection*** As in mammals, teleost fish classical MHC class II genes have been found linked with allograft rejection. [66] ***Indirect evidence for classical MHC class II function*** Although fragmentary, there is abundant evidence for helper and regulatory T cell functions in teleost fish similar to as in mammals. Furthermore, teleost fish have CD4, LAG-3 and CD74 molecules that probably all participate in the MHC class II system as in mammals. [31,32,59,60,67,68,69,70,71,72,73,74,75,76,77,78,79] **Functions of classical MHC class I** ***Expression of classical MHC class I*** As in mammals, teleost fish classical MHC class I transcripts and molecules are ubiquitously expressed and show the highest expression in epithelial and lymphoid tissues. Furthermore, as in mammals, their expression is upregulated after immune stimulation, which agrees with conservation of promoter motifs similar to those in mammals. Different from mammals is that in teleost fish, which are ectotherm species, the levels of MHC class I can be temperature dependent. [62,80,81,82,83,84] ***Peptide presentation*** As in mammals, teleost fish classical MHC class I molecules form heterotrimer complexes with β_2_-m and peptide ligands of ~9 aa. X-ray crystallography analysis revealed a similar complex structure as known in mammals. [85,86] ***Allograft rejection*** As in mammals, teleost fish classical MHC class I genes have been found linked with allograft rejection. [87,88] ***MHC class I restriction of cell-mediated cytotoxicity by T cells*** Although conclusive experiments have not been performed yet, there are several lines of evidence that together suggest that specific cell-mediated cytotoxicity by teleost fish CD8^+^ T cells requires classical MHC class I matching of the target cells as known in mammals. [89,90,91,92,93,94,95]

**Table 2:** List of genome-wide QTL studies in rainbow trout and Atlantic salmon that investigated disease resistance or other immune traits. QTL are listed as described in the respective references, with the most important QTK underlined; double underlining is used if among the important QTL one was found especially important. Red font (IB) and green font (II) indications are used to indicate chromosomes with the *IB* region and the classical MHC class II locus, respectively. Indications of (IB) or (II) in bold font refer to the respective locus being located within the confidence interval region mapped to only a part of the chromosome in the respective study, Italic font refers to them being located outside that confidence interval region, and if in normal font we were unable to asses that matter. The *IA* regions with classical MHC class I map to rainbow trout Chr. 18 and to Atlantic salmon Chr. 27, but those chromosomes were not found to harbor QTL in the listed studies.

**A) Disease Resistance/Immunity Related QTL Studies in Rainbow Trout**		
**Resistance against**	**QTL encoding linkage group**	**Reference**
Infectious pancreatic necrosis (IPN) virus	Chr. 3, Chr. 7, Chr. 8, Chr. 14 **(IB)**, Chr. 16, Chr-17 *(II)*, Chr. 20, Chr. 24, Chr. 27	Ozaki et al. 2007 [201]
	Chr. 14 **(IB)**, Chr. 16	Ozaki et al. 2001 [200]
Infectious hematopoietic necrosis (IHN) virus	Chr. 17 **(II)**	Khoo et al. 2004 [205]
Viral hemorrhagic septicemia (VHS) virus	Chr. 2, Chr. 3, Chr. 4, Chr. 5, Chr. 17 *(II)*, Chr. 24	Verrier et al. 2013 [207]
Cold water disease *Flavobacterium psychrophilum* (bacterium)	Chr. 2, Chr. 3, Chr. 7, Chr. 10, Chr. 17 *(II)*, Chr. 21, Chr. 24, Chr. 25, Chr. 26, Chr. 29	Fraslin et al. 2018 [206]
	Chr. 3, Chr. 5, Chr. 8, Chr. 10, Chr. 13, Chr. 15, Chr. 25	Vallejo et al. 2017 [208]
	Chr. 8, Chr. 19, Chr. 25	Liu et al. 2015 [209]
	Chr. 1, Chr. 6, Chr. 7, Chr. 8, Chr. 11, Chr. 12, Chr. 14 *(IB)*, Chr. 25	Palti et al. 2015 [202]
	Chr. 2, Chr. 3, Chr. 6, Chr. 8, Chr. 12, Chr. 13, Chr. 20	Vallejo et al. 2014 [210]
	Chr. 5, Chr. 16, Chr. 19	Wiens et al. 2013 [211]
*Ceratomyxa shasta* (parasite)	Chr. 9, Chr. 16, Chr. 20, Chr. 22, Chr. 29	Nichols et al. 2003 [212]
Whirling disease *Myxobolus cerebralis* (parasite)	Chr. 9	Baerwald et al. 2011 [213]
YAC-1 cells (murine tumor cell line)	Chr. 3	Zimmerman et al. 2004 [214]
**B) Disease Resistance/Immunity Related QTL Studies in Atlantic Salmon**		
Infectious pancreatic necrosis (IPN) virus	Chr. 26	Houston et al. 2010 [215]
	Chr. 4, Chr. 8, Chr. 26	Houston et al. 2008 [216]
	Chr. 1, Chr. 3, Chr. 4, Chr. 5, Chr. 6, Chr. 7, Chr. 9, Chr.10, Chr. 14 **(IB)**, Chr. 17, Chr. 18, Chr. 19, Chr. 20, Chr. 26	Moen et al 2009 [203]
Pancreas disease Salmonid alphavirus (SAV)	Chr. 2, Chr. 3, Chr. 4, Chr. 7, Chr. 14 (IB), Chr. 26	Gonen et al. 2015 [204]
Infectious Salmon Anaemia (ISA) virus	Chr. 15 (maybe additional weaker QTL, but difficult to interpret)	Moen et al. 2004 [217]
*Gyrodactylus salaris* (parasite)	Chr. 4, Chr. 5, Chr. 6, Chr. 10, Chr. 13, Chr. 15, Chr. 16, Chr. 17, Chr. 23, Chr. 24	Gilbey et al. 2006 [218]
